# The dynamic changes of flavor characteristics of sea cucumber (*Apostichopus japonicus*) during puffing revealed by GC–MS combined with HS-GC-IMS

**DOI:** 10.1016/j.fochx.2024.101709

**Published:** 2024-08-02

**Authors:** Xiaoqing Miao, Shuang Li, Yang Liu, Jing Li, Xiuping Dong, Ming Du, Pengfei Jiang

**Affiliations:** SKL of Marine Food Processing & Safety Control, National Engineering Research Center of Seafood, School of Food Science and Technology, Dalian Polytechnic University, Dalian 116034, China

**Keywords:** Sea cucumber, Puffing, HS-GC-IMS, GC–MS, Volatile organic compounds

## Abstract

To improve the ease of eating sea cucumbers, we investigated the impact of puffing temperature (190 °C - 250 °C) and time (1–5 min) on their quality and flavor. As temperature and time increased, sea cucumber puffing significantly enhanced. The microstructure of the puffed sea cucumber exhibited a uniform porous structure at 230 °C for 4 min. However, further puffing treatment caused the void to collapse. A total of 81 volatile organic compounds (VOCs) were identified using HS-GC-IMS, and 18 VOCs with Relative odor activity value (ROAV) ≥1 were identified. The content of fishy compounds, such as dimethyl sulfide, 1-octanal, and 1-nonanal in sea cucumbers gradually decreased with increasing temperature and time. Combined with GC–MS analysis indicating that the flavor of sea cucumbers puffed at 250 °C for 5 min was superior. Our findings suggest new avenues for sea cucumber processing and address the limited research on puffing techniques for protein-based raw materials.

## Highlights

• The puffing started earlier at higher temperatures.
• 81 VOCs were identified by GC-IMS, mainly including ketones and aldehydes.
• Dimethyl sulfide was the most significant VOC in all sea cucumber samples.
• Fishy dimethyl sulfide content reduced with increasing temperature and time.
• Sea cucumbers puffed at 250 °C for 5 min have a better flavor.

## 1 Introduction

Sea cucumber is a valuable marine invertebrate, with over 1200 known species worldwide, approximately 70 of which are edible (Gu et al., 2022; Pangestuti & Arifin, 2018; Xing, Sun, Liu, Zhang, & Yang, 2021). The body wall, rich in vitamins, minerals, and amino acids, is the edible part of the sea cucumber and is highly nutritious (García et al., 2019). Moreover, sea cucumbers contain active substances such as peptides, saponins, cerebrosides, and polysaccharides, which impart various physiological effects including anti-inflammatory, antitumor, and immune enhancement properties, making them valuable as tonic foods (Xu, Zhang, & Wen, 2018; Zhao, Xue, Zhang, & Wang, 2018). Currently, dried sea cucumber is one of the most important processed sea cucumber products in its original form (Liu et al., 2020). However, dried sea cucumbers require hydration prior to consumption (Liu et al., 2018). The rehydration process involves multiple soaking, washing, and boiling steps, which can be laborious and time-consuming, posing challenges to sea cucumber consumption. Therefore, leveraging existing processing technologies to address issues such as the complexity of pretreating dried sea cucumbers is crucial for enhancing their convenience.

Puffing is a physical processing technology based on phase change and gas thermal pressure effects, wherein rapid internal heating and vaporization cause the material to expand, resulting in a porous mesh tissue structure (Zapana et al., 2020). Puffing techniques commonly employed include extrusion puffing, hot air puffing, and microwave puffing, known for their simplicity, cost-effectiveness, and applicability to various raw materials (Choton, Gupta, Bandral, Anjum, & Choudary, 2020). Puffing can not only change the shape and structure of raw materials, but also the Maillard reaction produced during the puffing process can form new compounds and increase the flavor of raw materials (Zhang & Peterson, 2018).

Flavor is a crucial indicator of food quality (Chen et al., 2021). Headspace gas chromatography-ion mobility spectrometry (HS-GC-IMS) is a well-established technique for the determination of VOCs, combining the high-resolution capability of GC with the high discrimination of IMS, enabling rapid VOC detection in samples (Du, Chen, Liu, Wang, & Kong, 2021). HS-GC-IMS has shown great promise for applications in food adulteration, food detection, and food flavor analysis (Gerhardt, Birkenmeier, Sanders, Rohn, & Weller, 2017). Gas chromatography-mass spectrometry (GC–MS) is also widely used for volatile compound analysis in food, leveraging the separation and selectivity of GC and sensitivity and discrimination of MS for qualitative and quantitative analyses of complex compounds (Lee et al., 2014; Xu et al., 2020). Compared with HS-GC-IMS, GC–MS has a standardized reference database, but HS-GC-IMS is more efficient and easier to operate than GC–MS (Nie et al., 2022). Thus, integrating HS-GC-IMS and GC–MS can enhance the precision and sensitivity of flavor detection.

In this study, we analyzed the flavor differences in sea cucumbers under various puffing conditions using HS-GC-IMS and GC–MS. In addition, the effects of different puffing conditions on the microstructure of sea cucumbers were also explored by scanning electron microscopy (SEM). The findings from this study can provide certain reference value for the subsequent deep processing of sea cucumbers, filling crucial gaps in knowledge regarding the puffing of protein matrices.

## 2 Materials and methods

### 2.1 Materials and chemicals

Dried sea cucumbers were purchased from Dalian Xiaoqin Food Co., Ltd. They were washed and dried in an oven (SCC-WE101, Rational, Germany) at 90 °C for 30 min. Then the sea cucumbers were puffed at different temperatures (190 °C, 210 °C, 230 °C, 250 °C) for different times (1, 2, 3, 4, and 5 min). The puffed sea cucumbers were sealed and stored for further experiments. Sulfosalicylic acid was purchased from Shanghai Macklin Biochemical Technology Co., Ltd. (Shanghai, China). n-ketones (C4-C9) and common salts (all analytically pure) were obtained from Shanghai Aladdin Reagent Co., Ltd. (Shanghai, China).

### 2.2 Physical and structural characteristics of puffed sea cucumbers

#### 2.2.1 Puffing capability

The method described by Jia et al. (2020) was employed with minor adjustments. Puffed sea cucumber was placed in a graduated cylinder, filled with fine sand, gently tapped to occupy the pores, and the combined volume of sea cucumbers and sand was measured. The fine sand was then separated from the sea cucumber, and its volume was measured using the same cylinder. The disparity between the two volumes represents the volume of the puffed sea cucumber (V_2_). The same method was used to determine the volume of the dried sea cucumbers used for expansion (V_1_). The volume difference before and after puffing was calculated (ΔV).

#### 2.2.2 Cryo-scanning electron microscopy

Puffed sea cucumbers were uniformly sliced, immersed in liquid nitrogen, and subsequently moved to a cryogenic chamber (10–20 °C) under vacuum conditions. The samples were then crushed and held at −65 °C for 30 min to sublimate the water in the samples and gold plated for 60 s, and examined using a scanning electron microscope at a temperature of −140 °C, an accelerating voltage of 10.0 kV, and a magnification of 100× (SU8010, HITACHI, Japan) to analyze the microstructure of the puffed sea cucumbers.

### 2.3 Sensory analysis

The sensory evaluators consisted of 10 food professionals (5 females and 5 males). The five sensory attributes of puffed sea cucumber, including color, taste, morphology, umami and fishy were scored separately using a 9-point rule, and the scoring rules are shown in Table S1. The sensory experiment was approved by Dalian Polytechnic University and all participants participated voluntarily and signed a right to information.

### 2.4 Free amino acids (FAAs) assay

Free amino acids (FAAs) were extracted using the method proposed by Lin (2024). Accurately weigh 2 g of sea cucumber samples in a 50 mL volumetric flask, add water to fully dissolve the sample and then volume to the scale line, mix well and leave for 24 h. Aspirate 20 mL of the supernatant into a 10 mL centrifugal tube, add 20 ml of 5% sulphosalicylic acid solution to mix well and centrifugate the sample at 6000 g for 10 min. Aspirate 20 mL of supernatant and evaporate it to dryness in rotary evaporator and then add 1 mL sodium citrate buffer to dissolve the sample and then filter it through 0.45 μm membrane, and then analyze the sample by an amino acid analyzer (Biochrom 30+, Biochrom, England).

### 2.5 HS-GC-IMS analysis

The FlavourSpec® flavor analyzer (G.A.S., Dortmund, Germany) was used to detect volatile compounds in puffed sea cucumbers. The parameters of the method were slightly modified according to Xie et al. (2023) with some modifications. Puffed sea cucumber was broken and 0.5 g was accurately weighed into a 20 mL headspace vial with 4 mL of water and 0.2 g of amino acid (to inhibit interference from ammonia and trimethylamine), and incubated at a constant temperature of 60 °C, 500 r/min for 15 min. Subsequently, autosampling (500 μL) was performed using a high-temperature syringe (85 °C) in splitterless injection mode. VOCs were analyzed on a capillary column (MXT-WAX, 15 m × 0.53 mm, 1.0 μm, Restek, USA) with high-purity nitrogen as the carrier gas at a column temperature of 60 °C for 25 min. The programmed ramping parameters were as follows: the initial flow rate of 2.0 mL/min was maintained for 2 min, and then increased linearly to 10.0 mL/min within 8 min, 100.0 mL/min within 10 min, and 150.0 mL/min within 5 min. IMS conditions were as follows: tritium (3H) ionization source in positive ion mode, with a 98 mm drift tube length, drift tube temperature of 45 °C, and high-purity nitrogen as the drift gas at a flow rate of 150.0 mL/min.

### 2.6 GC–MS analysis

Referring to the method of Yin et al. (2020) with slight modification. A meteorological chromatograph (7890B, Agilent, USA) combined with a mass spectrometer (Pegasus BT, LECO, USA) was used to extract the VOCs in sea cucumbers. 2 g of sea cucumber was accurately weighed into a 20 mL headspace vial and incubated at a constant temperature of 50 °C for 15 min. DVB/CAR/PDMS (50/30 μm) SPME fiber (Supelco, Bellefonte, PA, USA) was aged at 260 °C for 10 min and then pulled out and inserted into the headspace vial to extract for 30 min. It was then transferred to the GC injection port and resolved at 250 °C for 5 min. The samples were analyzed on a DB-wax (30 m × 0.25 mm × 0.25 μm, Agilent, USA) chromatographic column for separation. The sample was injected in non-split mode, with helium as the carrier gas, at an injection temperature of 260 °C, a constant flow rate of 1 mL/min, and an initial temperature of 40 °C held for 5 min, and then the temperature increased to 220 °C at 5 °C/min, and then to 250 °C at 20 °C/min, retained for 2.5 min. Mass spectrometry was utilized by the full-scan method at a scanning rate of 10spec/s, an ionization energy of 70 eV, and a solvent delay time of 3 min.

### 2.7 Relative odor activity value (ROAV)

The flavor contributions of the VOCs were calculated by the ROAV method. The ingredient in the sample that contributed the most to the overall flavor was selected and denoted as ROAV_stan_ = 100. The ROAV of the other VOCs in the sample was calculated using the following formula (Li et al., 2023):

(1) $$\mathrm{ROAV}=\frac{C_{i}}{C_{\mathrm{stan}}}\times \frac{T_{\mathrm{stan}}}{T_{i}}\times 100$$

Where Ci (%) and Ti (μg/kg) represent the relative contents of VOCs in sea cucumbers, and Cstan (%) and Tstan (μg/kg) represent the relative contents and sensory thresholds of the VOCs with the highest contributions, respectively. The relative content of VOC was calculated by peak area normalization method. The thresholds for the VOCs are based on the book “Compendium of Odor Concentration Values in Air, Water, and Other Media (Second Expanded and Revised Edition)” and a number of references (Wei et al., 2023; Zhang et al., 2018).

### 2.8 Statistical analysis

Perform a minimum of three independent experiments for each trial. Data analysis was performed using SPSS 18.0 software (SPSS Inc., Chicago, IL, USA). VOCs were assessed using the software and plug-ins of the HS-GC-IMS instrument. Histograms were generated using GraphPad Prism. Heatmaps and Principal Component Analysis (PCA) were conducted using the online platform MetaboAnalyst 5.0.

## 3 Results and discussion

### 3.1 Physical properties of sea cucumbers under different puffing conditions

**Fig. 1:**
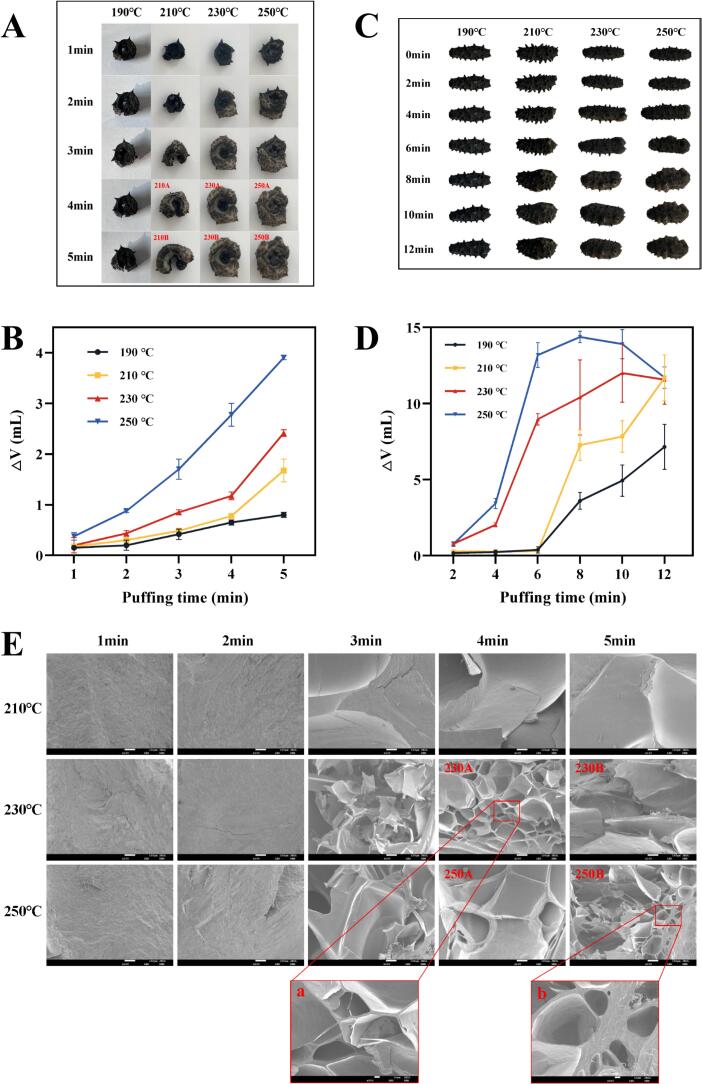
(A) Physical image of lump puffed sea cucumber. (B) Line graph of volume change of lump puffed sea cucumber. (C) Physical image of whole puffed sea cucumber. (D) Line graph of volume change of whole puffed sea cucumber. (E) Microstructure of puffed sea cucumber with 100× magnification. *E*-a and E-b were microstructures of puffed sea cucumbers magnified 500 × .

Fig. 1A showed the physical images of the sea cucumber samples under different puffing conditions, clearly illustrating significant changes in their appearance after puffing. There was no significant change in the volume of the sea cucumber puffed at 190 °C. The volume of the sea cucumber puffed at 210 °C showed significant changes after 3 min. The sea cucumbers puffed at 230 °C and 250 °C showed significant increase in volume after 2 min, indicating that the puffing started earlier at higher temperatures.

To demonstrate the change in sea cucumber volume under different puffing conditions, a line graph of volume change was plotted (Fig. 1B). This graph showed that the volume change of sea cucumber at 190 °C was not obvious, and it was consistent with the results of physical images. Additionally, the degree of volume change in sea cucumbers increased with the increase of puffing temperature and time. At 5 min, the volume puffing degree of all sea cucumber samples reached its maximum value, indicating the best puffing effect was achieved at 5 min. To further reveal the volume changes of sea cucumbers under different puffing conditions, we puffed the whole sea cucumber at 190 °C, 210 °C, 230 °C and 250 °C for 0, 2, 4, 6, 8, 10, and 12 min, respectively. The physical images of the whole puffed sea cucumber were shown in Fig. 1C. At 190 °C, again, no obvious change in the volume of the sea cucumber could be observed with the naked eye and at 210 °C, the sea cucumber started to puffed from 6 min, while at 230 °C and 250 °C, the sea cucumber started to puffed from 4 min. The results of whole sea cucumber puffing were consistent with those for lump sea cucumber, showing that puffing started earlier at higher temperatures. As shown in Fig. 1D, the degree of volume change of sea cucumbers at 190 °C and 210 °C both reached their maximum at 12 min. The maximum volume of the sea cucumber at 230 °C appeared at 10 min, and 250 °C, it appeared at 8 min. The puffing degree of the sea cucumbers treated at 230 °C and 250 °C began to decrease after reaching the maximum value, indicating that volume would no longer increase after a certain period, probably due to the high temperature and prolonged heating which caused the water in the sea cucumber to be severely evaporated, resulting in the volume shrinkage. The results for both lump and whole sea cucumbers proved that higher temperatures initiated puffing earlier and revealed that the volume of the sea cucumbers would not increase after a certain puffing time.

### 3.2 Microstructure characterization of sea cucumbers under different puffing conditions

It was essential to investigate the alterations in the microstructure of puffed sea cucumbers to gain a clearer understanding of the impact of puffing conditions on them. As can be seen from Fig. 1A, sea cucumbers were not puffed under the condition of 190 °C (1–5 min), Therefore, we focused on analyzing sea cucumber microstructures at 210 °C (1–5 min), 230 °C (1–5 min) and 250 °C (1–5 min), as shown in Fig. 1E. At different puffing temperatures, puffing for 1–2 min did not achieve complete puffing, with structural changes becoming evident after 3 min, consistent with the physical image observations. The puffed sea cucumbers appeared flaky at 210 °C (3–5 min). In contrast, at 230 °C (3–5 min) and 250 °C (3–5 min), they exhibited a hole structure. The main reason for this difference in microstructure was that with the increase in puffing temperature and time, water evaporation from the sea cucumbers were accelerated, and the water molecules absorbed heat and activated and converted into kinetic energy, which facilitated their movement between the cells and the cell walls at different speeds. This process eventually leads to the formation of multiple internal pore structures (Yang et al., 2020). Conversely, puffing at 210 °C failed to impart adequate kinetic energy to water, hindering the puffing of sea cucumber structure under these conditions. Notably, in 230 A the sea cucumber structure showed a uniform hole structure, and a 500× magnification of 230 A could reveal that the surface of the holes was smooth (Fig. 1E.a), and further puffing treatment would lead to larger holes and collapse. Pore collapse could be clearly seen in 250B, and a 500× magnification of the holes in 250B could be found to have a rougher surface (Fig. 1E.b). This indicated that longer puffing durations at higher temperatures caused crumpling of the sea cucumber microstructure. Based on the combined results of volume and microstructure experiments, we chose sea cucumber samples puffed at 230 °C for 4 min (230 A), 5 min (230B), and at 250 °C for 4 min (250 A), 5 min (250B) for the flavor investigation.

### 3.3 Sensory profiles of sea cucumbers under different puffing conditions

**Fig. 2:**
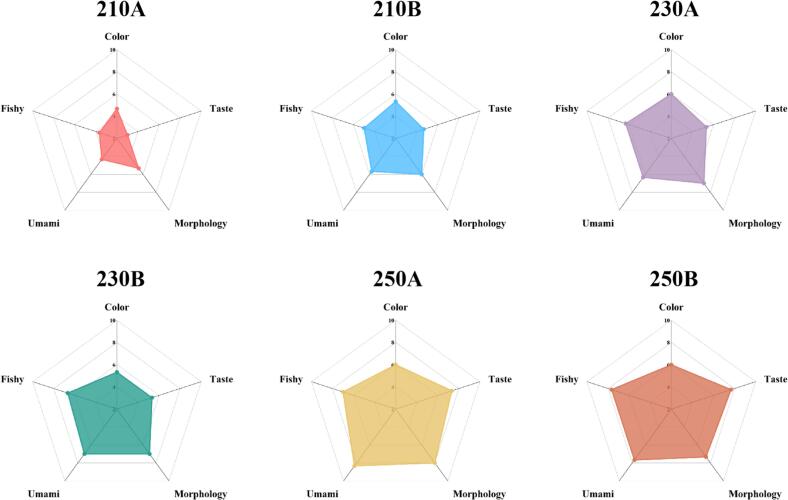
The sensory quality of sea cucumbers under different puffing conditions.

We performed sensory evaluation of five attributes of sea cucumbers with different expansion conditions: color, taste, morphology, umami and fishy, and the results are shown in Fig. 2. There were significant differences in the sensory profile of the six sea cucumber samples. Appearance is a direct factor affecting consumers' purchasing desire. In terms of color and morphology, 210 A and 210B had the lowest scores, suggesting that lower puffing temperatures would lead to uneven color and obvious cracks in the appearance of the sea cucumbers. In terms of taste, the scores tended to increase with increasing puffing temperature and time. 250 A and 250B had significantly higher taste scores than the other two groups. The sea cucumbers puffed at 250 °C had a crispy taste and could be chewed normally. In contrast, 210 A and 210B had the lowest scores. Lower puffing temperature and time would prevent the sea cucumber from puffing completely, which in turn resulted in a hard taste that could not be chewed properly. In terms of umami, 250 A had the highest score of 8.33, indicating that its umami odor was the most pronounced, followed by 250B. fishy odor is a major factor affecting the flavor of aquatic products. As the puffing temperature and time increased, the fishy odor score showed an increasing trend, indicating that the fishy odor of the sea cucumber was weakening. In conclusion sea cucumbers puffed at 250 °C had better flavor.

### 3.4 FAAs analysis

The types and contents of FAA confer unique flavor characteristics to sea cucumbers. As shown in Table 1, 17 FAAs were detected in all sea cucumbers under different puffing conditions, mainly including three major categories of fresh, sweet, and bitter amino acids, which brought rich flavors to sea cucumbers. However, the content of each FAA differed significantly, and the FAA with higher contents in six sea cucumbers were glutamic acid (Glu), cysteine (Cys) and arginine (Arg), with Cys being significantly more abundant than the other FAAs. (See Table 1.)

**Table 1:** Free amino acid content in sea cucumbers under different puffing conditions (mg/100 g).

FAAs	Flavor characteristics	210 A	210B	230 A	230B	250 A	250B
Asp	Umami	2.85 ± 0.07^ab^	2.55 ± 0.64^ab^	2.75 ± 0.35^ab^	2.15 ± 0.21^b^	3.40 ± 0.28^ab^	3.70 ± 0.14^a^
Glu	Umami	27.50 ± 1.98^a^	27.70 ± 0.99^a^	20.30 ± 0.99^c^	23.90 ± 0.99^b^	26.40 ± 0.57^ab^	20.55 ± 0.21^c^
Thr	sweetness	1.35 ± 0.21^a^	1.50 ± 0.28^a^	1.35 ± 0.21^a^	1.00 ± 0.00^a^	1.90 ± 0.14^a^	1.65 ± 0.35^a^
Ser	sweetness	1.90 ± 0.14^a^	2.00 ± 0.00^a^	2.00 ± 0.14^a^	1.75 ± 0.35^a^	2.15 ± 0.07^a^	2.50 ± 0.28^a^
Gly	sweetness	0.50 ± 0.00^b^	0.50 ± 0.14^b^	0.70 ± 0.28^ab^	1.40 ± 0.14^a^	1.25 ± 0.35^ab^	1.15 ± 0.07^ab^
Ala	sweetness	3.40 ± 0.42^a^	3.00 ± 0.28^a^	3.35 ± 0.35^a^	3.50 ± 0.71^a^	4.50 ± 0.00^a^	3.55 ± 0.49^a^
Pro	bitterness	1.80 ± 0.14^a^	1.85 ± 0.07^a^	1.60 ± 0.14^a^	1.30 ± 0.28^a^	1.60 ± 0.14^a^	1.40 ± 0.00^a^
Cys	bitterness	29.00 ± 0.28^a^	28.35 ± 1.06^a^	28.65 ± 0.92^a^	28.30 ± 0.57^a^	26.95 ± 1.34^a^	29.00 ± 1.27^a^
Val	bitterness	5.70 ± 0.14^a^	5.20 ± 0.28^a^	5.30 ± 0.71^a^	6.60 ± 0.28^a^	5.40 ± 1.13^a^	4.75 ± 0.21^a^
Met	bitterness	3.00 ± 0.85^a^	2.75 ± 0.21^a^	4.00 ± 0.28^a^	4.15 ± 0.64^a^	3.20 ± 0.00^a^	2.85 ± 0.07^a^
Ile	bitterness	0.65 ± 0.07^c^	2.20 ± 0.42^b^	1.70 ± 0.14^b^	2.35 ± 0.35^b^	1.55 ± 0.21^b^	3.50 ± 0.00^a^
Leu	bitterness	0.95 ± 0.07^a^	0.60 ± 0.00^a^	0.75 ± 0.07^a^	0.40 ± 0.00^a^	0.65 ± 0.49^a^	0.95 ± 0.07^a^
Tyr	bitterness	2.05 ± 0.78^b^	2.30 ± 0.00^b^	2.60 ± 0.57^b^	3.15 ± 0.49^b^	4.80 ± 0.14^a^	2.55 ± 0.35^b^
Phe	bitterness	1.80 ± 0.57^a^	0.85 ± 0.21^a^	0.95 ± 0.07^a^	1.05 ± 0.49^a^	1.10 ± 0.14^a^	0.90 ± 0.14^a^
His	bitterness	1.10 ± 0.57^a^	1.15 ± 0.07^a^	0.60 ± 0.28^a^	0.85 ± 0.64^a^	0.75 ± 0.07^a^	0.65 ± 0.07^a^
Lys	bitterness	2.00 ± 0.14^a^	1.50 ± 0.14^ab^	1.25 ± 0.07^ab^	0.70 ± 0.42^b^	1.15 ± 0.21^ab^	1.05 ± 0.49^ab^
Arg	bitterness	13.85 ± 0.07^b^	14.70 ± 0.14^a^	12.55 ± 0.21^c^	12.60 ± 0.00^c^	14.60 ± 0.28^a^	13.00 ± 0.14^c^
TEAAs		99.40 ± 0.42^b^	98.70 ± 0.14^c^	90.40 ± 0.14^f^	95.15 ± 0.35^d^	101.35 ± 0.07^a^	93.70 ± 0.28^e^

TFAAs: total FAAs content.

The total FAA content in sample 250 A was significantly higher than that in the other sea cucumbers, while 250B had the lowest total FAA content. This difference may be due to the degradation of more proteins into FAAs under high temperatures. As puffing time increased, more FAAs began to decompose, producing a large number of flavor components, which led to a decrease in FAA content. When the puffing time was 5 min, the total FAA content decreased with increasing puffing temperature, which indicated that the increase of temperature could induce more FAA to have a Maillard reaction with carbohydrates (Wang, Yang, Zhao, Zhang, & Su, 2019), leading to a reduction in FAA content.

### 3.5 HS-GC-IMS analysis

#### 3.5.1 HS-GC-IMS topographic plots of sea cucumbers under different puffing conditions

**Fig. 3:**
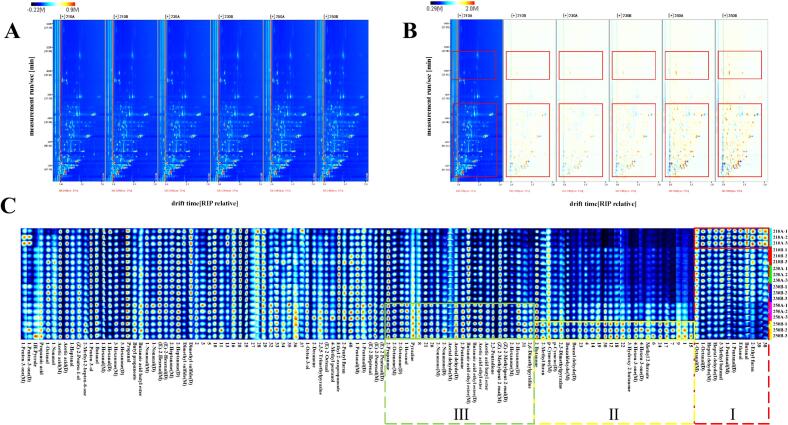
GC-IMS analysis of VOCs in sea cucumbers under different puffing conditions. (A) Spectra of volatile compositions (top view). (B) Comparative difference spectra of volatile compositions. (C) Fingerprinting of sea cucumbers under different puffing conditions.

HS-GC-IMS was used to identify the VOCs in sea cucumbers under different puffing conditions. The two-dimensional spectra of VOCs in sea cucumbers were obtained by projecting the three-dimensional spectra, as shown in Fig. 3A. Each point on the right side of the ion peak represents a volatile compound (Zheng et al., 2022). Most of the signals in the sea cucumber samples appeared in the range of retention time 200–1000 s and drift time 1.0–2.0 s. Using the spectrum of 210 A as a reference, and subtracting the spectra of the other samples as a reference when the concentrations are the same, the subtracted background is white, as shown in Fig. 3B. It is obvious that there was a significant difference between the VOCs in 250 A and 250B compared to the other sea cucumber samples, which had the reddest signals, indicating that the content was higher than other sea cucumber samples. At identical puffing temperatures, sea cucumbers subjected to a 5-min puffing duration displayed a markedly greater intensity of red signals compared to those puffed for 4 min, implying an augmentation in VOC content with prolonged puffing durations.

#### 3.5.2 Fingerprint analysis of VOCs from puffed sea cucumbers

The targets were characterized using the NIST 2020 database and the IMS migration time databases (Wang et al., 2022). As shown in Table 2, HS-GC-IMS clearly characterized 81 VOCs (monomers and dimers of some substances), mainly including 25 aldehydes, 12 alcohols, 21 ketones, 8 esters, 3 acids, 2 benzene rings, 2 sulfides, and 8 heterocycles, indicating a rich composition of flavor components in sea cucumbers that provide their unique flavor.

**Table 2:** Information about VOCs in puffed sea cucumbers identified by GC-IMS.

NO.	Compound	CAS	Formula	RI	RT	DT
1	1-Nonanol	C143088	C_9_H_20_O	1619.6	1312.829	1.5347
2	1-Octanol	C111875	C_8_H_18_O	1548.5	1132.979	1.46474
3	Propanoic acid	C79094	C_3_H_6_O_2_	1536	1104.17	1.10113
4	1H-Pyrrole	C109977	C_4_H_5_N	1491.6	1007.032	0.97958
5	Benzaldehyde(M)	C100527	C_7_H_6_O	1477.3	977.638	1.15601
6	Benzaldehyde(D)	C100527	C_7_H_6_O	1476.9	976.821	1.47713
7	Acetic acid(M)	C64197	C_2_H_4_O_2_	1448.6	921.299	1.05368
8	Acetic acid(D)	C64197	C_2_H_4_O_2_	1448.6	921.299	1.14719
9	1-Heptanol	C111706	C_7_H_16_O	1440.9	898.816	1.39761
10	2-Nonanone(M)	C821556	C_9_H_18_O	1394.4	772.34	1.41063
11	2-Nonanone(D)	C821556	C_9_H_18_O	1396.3	775.316	1.88728
12	1-Hexanol	C111273	C_6_H_14_O	1367.7	731.155	1.32801
13	(Z)-2-Penten-1-ol	C1576950	C_5_H_10_O	1328.7	674.728	0.95039
14	2-Methyl-2-hepten-6-one	C110930	C_8_H_14_O	1350.1	705.12	1.18136
15	(E)-2-Heptenal	C18829555	C_7_H_12_O	1332.1	679.474	1.26077
16	1-Octanal(M)	C124130	C_8_H_16_O	1294.4	628.348	1.40769
17	1-Octanal(D)	C124130	C_8_H_16_O	1295.4	630.151	1.83015
18	2-Octanone(M)	C111137	C_8_H_16_O	1290.1	619.33	1.33169
19	2-Octanone(D)	C111137	C_8_H_16_O	1292	623.298	1.76575
20	3-Hydroxy-2-butanone	C513860	C_4_H_8_O_2_	1280.2	599.131	1.05735
21	1-Penten-3-ol	C616251	C_5_H_10_O	1168.9	411.081	0.95153
22	4-Hexen-3-one(M)	C2497214	C_6_H_10_O	1205.9	467.621	1.11323
23	4-Hexen-3-one(D)	C2497214	C_6_H_10_O	1205	466.092	1.4665
24	(Z)-2-Methylpent-2-enal(M)	C623369	C_6_H_10_O	1158.8	396.435	1.16211
25	(Z)-2-Methylpent-2-enal(D)	C623369	C_6_H_10_O	1158.8	396.435	1.50332
26	1-Butanol	C71363	C_4_H_10_O	1154.2	389.994	1.18439
27	(E)-2-Pentenal(M)	C1576870	C_5_H_8_O	1142.4	374.022	1.10678
28	(E)-2-Pentenal(D)	C1576870	C_5_H_8_O	1142.7	374.376	1.36919
29	1-Hexanal(M)	C66251	C_6_H_12_O	1097.9	319.074	1.26019
30	1-Hexanal(D)	C66251	C_6_H_12_O	1097.7	318.849	1.57001
31	2-Hexanone(M)	C591786	C_6_H_12_O	1092.7	314.077	1.19612
32	2-Hexanone(D)	C591786	C_6_H_12_O	1094.3	315.529	1.50396
33	3-Hexanone(M)	C589388	C_6_H_12_O	1065.9	290.079	1.17254
34	3-Hexanone(D)	C589388	C_6_H_12_O	1064.8	289.111	1.48196
35	2,3-Pentadione	C600146	C_5_H_8_O_2_	1059.1	284.27	1.21169
36	Butanoic acid ethyl ester(M)	C105544	C_6_H_12_O_2_	1047.4	274.587	1.20285
37	Butanoic acid ethyl ester(D)	C105544	C_6_H_12_O_2_	1047.8	274.91	1.57162
38	1-Pentanal(M)	C110623	C_5_H_10_O	994.4	234.864	1.18291
39	1-Pentanal(D)	C110623	C_5_H_10_O	993.5	234.306	1.42921
40	2-Pentanone	C107879	C_5_H_10_O	991.8	233.331	1.37197
41	Ethanol	C64175	C_2_H_6_O	943	206.154	1.12429
42	2-Butanone	C78933	C_4_H_8_O	911.1	190.17	1.24996
43	3-Methyl butanal	C590863	C_5_H_10_O	926.1	197.547	1.41464
44	Acetic acid ethyl ester	C141786	C_4_H_8_O_2_	890.1	180.334	1.3427
45	Butanal	C123728	C_4_H_8_O	881.2	176.292	1.28806
46	2-Methylfuran	C534225	C_5_H_6_O	872.2	172.295	0.9875
47	2-Propanone	C67641	C_3_H_6_O	830.4	154.982	1.12427
48	Propanal	C123386	C_3_H_6_O	804.9	145.286	1.15044
49	Pyrazine	C290379	C_4_H_4_N_2_	1220.1	490.28	1.05221
50	Butyl propanoate	C590012	C_7_H_14_O_2_	1149.5	383.505	1.28853
51	Butanoic acid butyl ester	C109217	C_8_H_16_O_2_	1222.1	493.6	1.3328
52	1-Nonanal(M)	C124196	C_9_H_18_O	1398.2	778.497	1.47235
53	1-Nonanal(D)	C124196	C_9_H_18_O	1398.6	779.798	1.95585
54	1-Pentanol(M)	C71410	C_5_H_12_O	1259.5	559.271	1.25692
55	1-Pentanol(D)	C71410	C_5_H_12_O	1262	563.809	1.51626
56	p-Cymene(M)	C99876	C_10_H_14_	1263.3	566.284	1.29815
57	p-Cymene(D)	C99876	C_10_H_14_	1260.4	560.921	1.72906
58	(E)-2-Hexenal(M)	C6728263	C_6_H_10_O	1226.2	500.272	1.18643
59	(E)-2-Hexenal(D)	C6728263	C_6_H_10_O	1226.9	501.51	1.5269
60	Heptaldehyde(M)	C111717	C_7_H_14_O	1192.2	446.638	1.32874
61	Heptaldehyde(D)	C111717	C_7_H_14_O	1195	450.764	1.70645
62	2-Heptanone(M)	C110430	C_7_H_14_O	1185.4	435.911	1.26357
63	2-Heptanone(D)	C110430	C_7_H_14_O	1188	440.037	1.64261
64	1-Penten-3-one(M)	C1629589	C_5_H_8_O	1035.3	264.904	1.07308
65	1-Penten-3-one(D)	C1629589	C_5_H_8_O	1033.5	263.513	1.32139
66	Dimethyl sulfide(M)	C75183	C_2_H_6_S	785.6	138.352	0.9642
67	Dimethyl sulfide(D)	C75183	C_2_H_6_S	780.7	136.663	1.12465
68	Acetaldehyde(M)	C75070	C_2_H_4_O	732.3	120.902	0.96347
69	Acetaldehyde(D)	C75070	C_2_H_4_O	745	124.842	1.13931
70	Methyl 2-furoate	C611132	C_6_H_6_O_3_	1509.8	1045.745	1.15235
71	1-Octen-3-ol	C3391864	C_8_H_16_O	1438.2	890.532	1.16587
72	2-Decanone	C693549	C_10_H_20_O	1499	1022.615	1.46545
73	2,3,5-Trimethylpyrazine	C14667551	C_7_H_10_N_2_	1423.8	848.519	1.16654
74	(E)-2-Octenal	C2548870	C_8_H_14_O	1416.5	828.073	1.34088
75	2,3-Dimethylpyrazine	C5910894	C_6_H_8_N_2_	1368.1	731.674	1.12074
76	4-Methyl pentanol	C626891	C_6_H_14_O	1308.9	647.891	1.30971
77	2,6-Dimethylpyridine	C108485	C_7_H_9_N	1273.2	585.296	1.08536
78	Ethyl 2-oxopropanoate	C617356	C_5_H_8_O_3_	1251.1	543.769	1.15549
79	2-Pentyl furan	C3777693	C_9_H_14_O	1236.5	517.926	1.25939
80	2-Ethyl furan	C3208160	C_6_H_8_O	962.9	216.843	1.05192
81	Acetic acid butyl ester	C123864	C_6_H_12_O_2_	1080.8	303.197	1.24786

M, D: monomer, dimerization of the same substance, respectively.

The fingerprint profiles of the VOCs in sea cucumbers under different puffing conditions, as shown in Fig. 3C. 2-Ethylfuran, 1-pentanal(D), 3-methyl butanal, heptaldehyde, and octanal exhibited the highest content in 210 A, demonstrating a declining trend with rising puffing temperature (Fig. 3C-I). These compounds typically emit a fishy odor in aquatic products, suggesting a decline in the fishy aroma of sea cucumbers with rising puffing temperature. Methyl 2-furoate (fruity odor), 4-hexen-3-one (fruity odor), 3-hydroxy-2-butanone (sweetness), 2-methylfuran (nutty odor), and 2-butanone (butterscotch odor) were the most abundant in 250B, while they were less abundant in other sea cucumbers, and their contents showed a decreasing tendency with the increase of the puffing temperature (Fig. 3C-II), giving 250B a pleasing flavor. 2-Hexanone (fruity odor), 2,3-pentadione (cheese odor), acetic acid butyl ester (fruity odor), acetic acid ethyl ester (fruity odor), and 1-hexanol (fruity odor) were higher, and similar in 250 A and 250B, and the content of these components showed an increasing trend with increasing temperature (Fig. 3C-III), indicating that the fruity flavor of sea cucumbers puffed at 250 °C was stronger and the flavor was better. This indicated that increasing the puffing temperature played an essential effect on improving the flavor of sea cucumbers. In addition, acetic acid(M), 1-penten-3-ol, and propanal were found in high levels in all the sea cucumbers, which were hypothesized to be the major VOCs in sea cucumbers. 1-Penten-3-ol and propanal are components commonly found in aquatic products, while the higher levels of acetic acid(M) were attributed to the higher salt content in dried sea cucumbers.

#### 3.5.3 Differences in VOCs of sea cucumbers under different puffing conditions

**Fig. 4:**
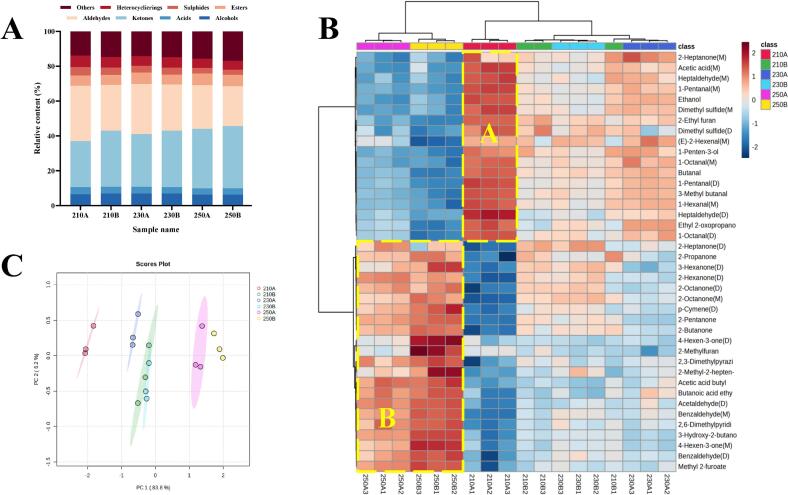
(A) Relative content of VOCs species in sea cucumbers under different puffing conditions by GC-IMS. (B) Heatmap of TOP 40 VOCs in sea cucumbers under different puffing conditions by GC-IMS. (C) PCA analysis of GC-IMS.

The relative contents of VOCs in the six sea cucumber samples were determined based on peak volumes of VOCs (Table S2), and the percentage of each type of substance was plotted in Fig. 4A. Ketones and aldehydes were relatively predominant in the sea cucumber samples, comprising approximately 58% of the total VOC content. This finding aligned with previous studies on VOCs in sea cucumbers (Zhang et al., 2018). With consistent puffing temperatures, ketone levels increased, whereas aldehyde levels decreased with prolonged puffing time. However, the combined proportion of both types remained at approximately 58%. Ketones primarily originated from amino acid breakdown and the thermal oxidative degradation of polyunsaturated fatty acids, whereas aldehydes stemmed from lipid oxidation and degradation (Xu et al., 2023; Zeng et al., 2020), However, because ketones have a higher threshold compared to aldehydes, their contribution to the overall flavor of puffed sea cucumbers was less significant than that of aldehydes.

The top 40 VOCs were selected to generate heatmaps (Fig. 4B). According to the vertical pattern, the six sea cucumber samples could be classified into two major groups, one group included 250 A and 250B, and the other group included 210 A, 210B, 230 A and 230B. This classification indicated that the flavors of sea cucumbers puffed at 250 °C were more distinct from those of the other samples. These VOCs in zone A were most abundant in 210 A, and these VOCs generally showed a certain fishy odor in aquatic products. The contents of these VOCs decreased with increasing puffing temperature and time, and their levels at 250 °C were significantly lower than in the other groups. Heptaldehyde (M) was the most abundant volatile component in 210 A and conferred a fishy odor and clam flavor to the sea cucumbers. VOCs in zone B were most abundant in 250B, particularly 4-hexen-3-one (D), 2-methylfuran, and 2-methyl-2-hepten-6-one, which were significantly higher than in other sea cucumber samples. Most VOCs in zone B increased in content with higher puffing temperatures and longer puffing times. Many of these VOCs produced a creamy and fruity sea cucumber aroma, whereas some VOCs with larger threshold values had a smaller effect on the overall flavor. The heatmap indicated that the fishy flavor of puffed sea cucumbers gradually decreased with increasing puffing temperature and time, while pleasant aromas such as creamy and fruity aroma gradually increased.

#### 3.5.4 PCA analysis

The PCA results of sea cucumbers treated with different puffing conditions was shown in Fig. 4C. The first two principal components contributed 83.8% and 6.2%, respectively, with a total contribution rate of 90%. There was no overlap of sea cucumber samples at different puffing times at the same temperature, indicating that puffing time significantly affected the flavor of sea cucumbers. At the same temperature, the samples in the PCA were distributed from left to right as puffing times increased. Notably, 210B and 230B had overlapping areas, suggesting their flavors were more similar. Additionally, 210 A, 210B, 230 A and 230B were located in the left quadrant of the PCA, while 250 A and 250B were located in the right quadrant, with larger distances from the other groups. This indicated that the flavors of 250 A and 250B were significantly different from those of the other samples.

#### 3.5.5 Key VOCs in sea cucumbers under different puffing conditions

**Fig. 5:**
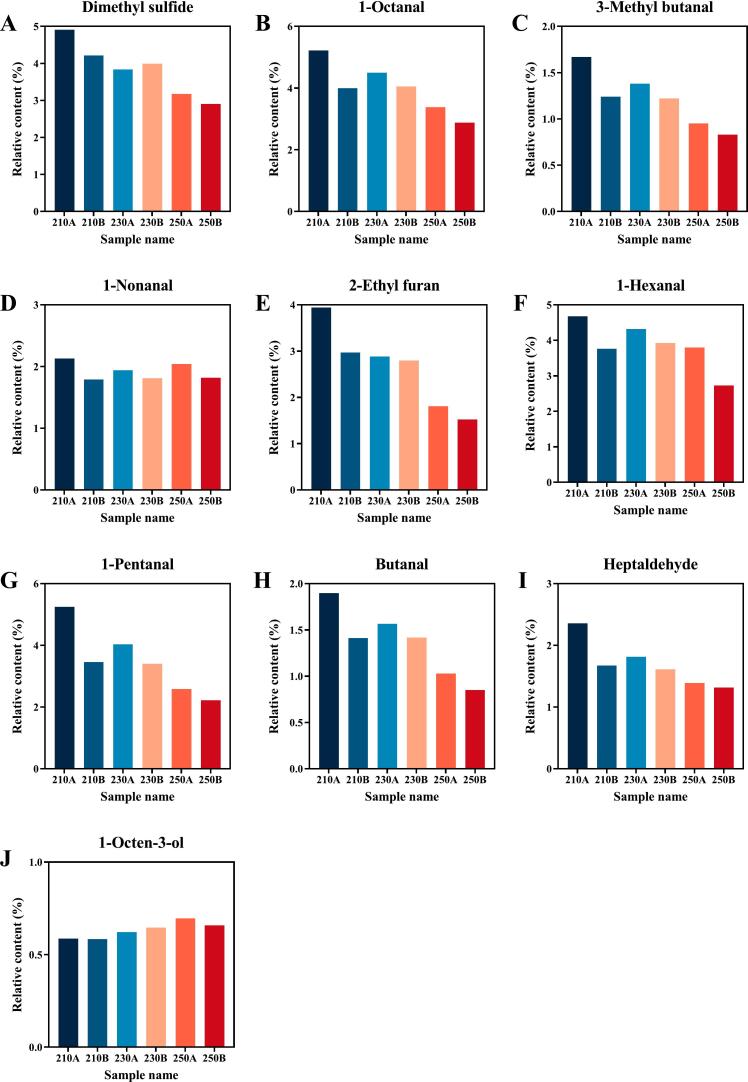
10 VOCs with ROAV ≥1 shared in sea cucumbers under different puffing conditions.

The key VOCs in sea cucumbers under different puffing conditions were characterized by ROAV, with higher values indicating a greater contribution of the VOCs to the flavor of sea cucumbers (Li et al., 2023). A total of 18 key VOCs were identified in six puffed sea cucumber samples (ROAV ≥1) (Table S3), with 10 key VOCs common to all samples (Fig. 5). Dimethyl sulfide was the most significant VOC in all the sea cucumber samples. and Li et al. (2022) also confirmed that dimethyl sulfide is one of the main sources of fishy flavor in seafood. The relative content of dimethyl sulfide decreased with increasing puffing temperature and time, indicating a reduction in the fishy flavor. In addition, 1-octanal, 3-ethyl butanal, 1-nonanal, and 2-ethyl furan contributed more to the overall flavor of the puffed sea cucumbers (Fig. 5). The contents of these four key VOCs showed the same trend as dimethyl sulfide, showing a negative correlation with puffing temperature and time. 1-Octanal, 3-methyl butanal, and 1-nonanal proved to be the main cause of the fishy odor (Zhou, Chong, Ding, Gu, & Liu, 2016). 2-Ethyl furan, an oxidation product of unsaturated fatty acids, imparts rubbery, pungent, and other off-flavors (Sun et al., 2023). Thus, the fishy and off-flavors of puffed sea cucumbers diminish with increasing puffing temperature and time.

### 3.6 Screening of key differential VOCs in sea cucumber under different puffing conditions based on GC–MS

**Fig. 6:**
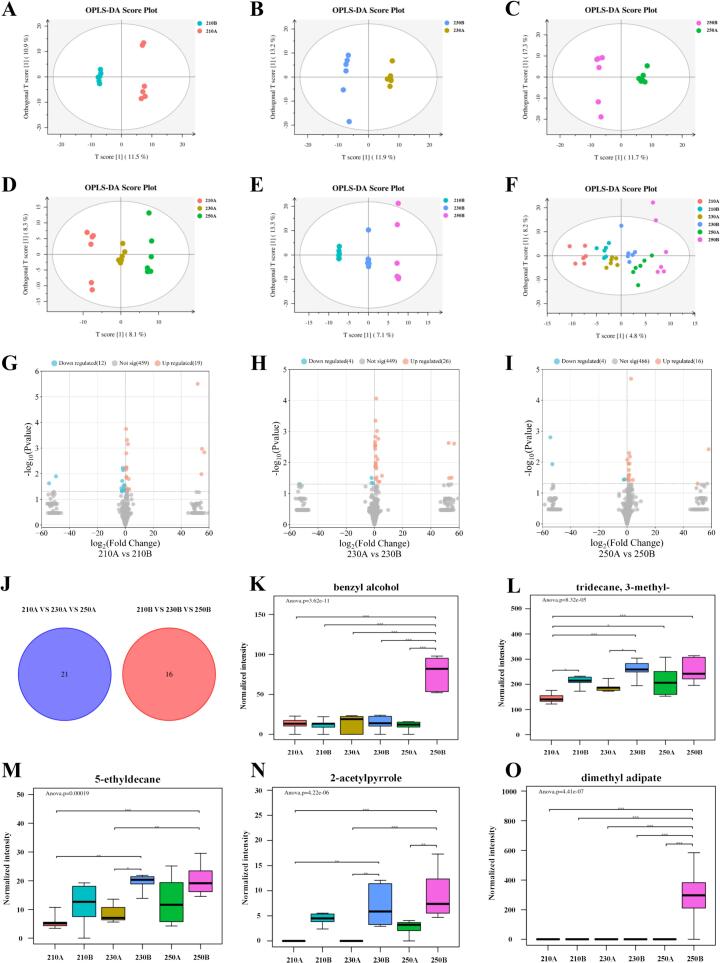
(A-F) Score plot of OPLS-DA. (J) VEEN plots of key differential VOCs in sea cucumbers puffed for 4 min and 5 min at different temperatures. (L-O) Box plots of the top 5 major differential VOCs of VIP in puffed sea cucumbers.

Comparison of the sea cucumber samples under different puffing conditions using OPLS-DA, respectively, was shown in Fig. 6A-F. In the six sets of data models, two samples with different times at the same temperature showed obvious separation trends, as did three samples with different temperatures. Additionally, there was no overlap between the six sea cucumber samples, and the R^2^X, R^2^Y and Q^2^ were 0.13, 0.952 and 0.309, respectively, which also proved that the constructed model could explain the data well.

Based on the OPLS-DA results, the key differential VOCs were using fold change (FC) > 1, variable importance in projection (VIP) > 1, and *p*-value <0.05. The volcano plot showed the comparison results of key differential VOCs between the two samples with different puffing times at the same puffing temperature (Fig. 6G-I). In the comparison between 210 A and 210B, 31 key differences in VOCs were identified, with 19 components upregulated and 12 downregulated. Similarly, in the comparison of 230 A and 230B, a total of 30 key differential VOCs were detected, with 26 components upregulated and 4 downregulated. In the comparison between 250 A and 250B, 20 key differences in VOCs were found, with 16 components upregulated and 4 downregulated. The volcano plot results suggested that sea cucumbers puffed for 5 min at the same temperature and had a richer flavor. VEEN plots for the two sample groups with different puffing temperatures at the same puffing time (Fig. 6J) revealed no key differences in VOCs between the groups, indicating significant flavor disparities between sea cucumbers puffed for 4 and 5 min.

The top 5 VIP values, including benzyl alcohol (VIP = 2.84), tridecane, 3-methyl- (VIP = 2.83), 5-ethyldecane (VIP = 2.83), 2-acetylpyrrole (VIP = 2.66), and dimethyl adipate (VIP = 2.63), were selected for box plot analysis (Fig. 6L-O). Benzyl alcohol, known for its toasty, rosy, and sweet flavors, exhibited significantly higher levels in 250B compared to the other sea cucumber samples, imparting a pleasant aroma to 250B (Fig. 6K). Tridecane, 3-methyl-, and 5-ethyldecane showed a gradual increase in concentration with prolonged puffing time (Fig. 6L-M). Although alkanes have a high threshold and minimal direct impact on sea cucumber flavor, their synergistic effect enhances the overall flavor profile. 2-Acetylpyrrole contributed typical baking aroma, caramel, nutty, and sweet flavors, with levels increasing with puffing duration, particularly in 250B (Fig. 6N). The puffing temperature also affected 2-acetylpyrrole content, with higher temperatures resulting in higher levels for the same puffing duration. This indicated that increasing the puffing temperature and time facilitate 2-acetylpyrrole formation, which was attributed to enhanced Maillard and caramelization reactions under high temperatures, leading to an increased pyrrole compound content (Yang et al., 2023). Dimethyl adipate, with its sweet, fruity aroma, was predominantly found in 250B (Fig. 6O), contributing to the softening of the overall flavor of sea cucumbers. These five key differential VOCs were notably abundant in 250B, presenting pleasant aromas, consistent with HS-GC-IMS results, indicating that sea cucumbers puffed at 250 °C for 5 min and exhibited superior flavor.

## 4 Conclusions

In this study, the effects of different puffing conditions on the structure and flavor of dried sea cucumbers were analyzed. It was found that the puffing of sea cucumbers was not significant at 190 °C. The sea cucumbers started to puff at 210 °C, 230 °C, and 250 °C at 3 min, 2 min and 2 min respectively. SEM showed that 2 min of puffing had little effect on the microstructure of the sea cucumbers, but after 3 min, the sea cucumbers at 210 °C exhibited a lamellar structure, while those at 230 °C and 250 °C showed a hole structure. 230 A showed a homogeneous multi-hole structure, but further puffing led to hole collapse. Glu, Cys and Arg were the FAAs with high content in puffed sea cucumber, with the highest TEAA found at 250 °C for 4 min. HS-GC-IMS clearly characterized 81 VOCs, of which 18 VOCs had ROAV ≥1. The contents of fishy components, such as dimethyl sulphide, 1-octanal, and 1-nonenal gradually decreased with increasing puffing temperature and time, indicating that fishy odor of sea cucumbers was weakening. Combined with the GC–MS results, the analysis indicated that the sea cucumber puffed at 250 °C for 5 min had the weakest fishy odor and better flavor. Both OPLS-DA and PCA results indicated that the sea cucumbers with different puffing conditions could be clearly distinguished from each other. The application of puffing technology to sea cucumbers provides a new approach to solving the difficulty of consuming sea cucumbers.

## CRediT authorship contribution statement

**Xiaoqing Miao:** Writing – review & editing, Writing – original draft, Formal analysis, Data curation, Conceptualization. **Shuang Li:** Software, Data curation. **Yang Liu:** Software, Data curation. **Jing Li:** Methodology, Investigation. **Xiuping Dong:** Visualization, Validation, Supervision. **Ming Du:** Visualization, Validation, Supervision. **Pengfei Jiang:** Writing – review & editing, Writing – original draft, Supervision, Resources, Conceptualization.

## Declaration of competing interest

The authors declare that they have no known competing financial interests or personal relationships that could have appeared to influence the work reported in this paper.

## Data Availability

No data was used for the research described in the article.

## Appendix A Supplementary data

Supplementary material Image 1

## References

[bb0005] Chen Y., Li P., Liao L., Qin Y., Jiang L., Liu Y. (2021). Characteristic fingerprints and volatile flavor compound variations in Liuyang Douchi during fermentation via HS-GC-IMS and HS-SPME-GC-MS. Food Chemistry.

[bb0010] Choton S., Gupta N., Bandral J.D., Anjum N., Choudary A. (2020). Extrusion technology and its application in food processing: A review. The Pharma Innovation.

[bb0020] Du H., Chen Q., Liu Q., Wang Y., Kong B. (2021). Evaluation of flavor characteristics of bacon smoked with different woodchips by HS-SPME-GC-MS combined with an electronic tongue and electronic nose. Meat Science.

[bb0025] García J., Méndez D., Álvarez M., Sanmartin B., Vázquez R., Regueiro L., Atanassova M. (2019). Design of novel functional food products enriched with bioactive extracts from holothurians for meeting the nutritional needs of the elderly. Lwt.

[bb0030] Gerhardt N., Birkenmeier M., Sanders D., Rohn S., Weller P. (2017). Resolution-optimized headspace gas chromatography-ion mobility spectrometry (HS-GC-IMS) for non-targeted olive oil profiling. Analytical and Bioanalytical Chemistry.

[bb0035] Gu P., Qi S., Zhai Z., Liu J., Liu Z., Jin Y., Wang F. (2022). Comprehensive proteomic analysis of sea cucumbers (Stichopus japonicus) in thermal processing by HPLC-MS/MS. Food Chemistry.

[bb0040] Jia F., Yang S., Ma Y., Gong Z., Cui W., Wang Y., Wang W. (2020). Extraction optimization and constipation-relieving activity of dietary fiber from Auricularia polytricha. Food Bioscience.

[bb0045] Lee H., Lim N., Cho K., Yang H., Yim K., Kim M., Jung W. (2014). Characterisation of inorganic elements and volatile organic compounds in the dried sea cucumber Stichopus japonicus. Food Chemistry.

[bb0050] Li Y., Yuan L., Liu H., Liu H., Zhou Y., Li M., Gao R. (2023). Analysis of the changes of volatile flavor compounds in a traditional Chinese shrimp paste during fermentation based on electronic nose, SPME-GC-MS and HS-GC-IMS. Food Science and Human Wellness.

[bb0060] Liu Y., Zhou D.Y., Liu Z., Lu T., Song L., Li D., Shahidi F. (2018). Structural and biochemical changes in dermis of sea cucumber (Stichopus japonicus) during autolysis in response to cutting the body wall. Food Chemistry.

[bb0065] Liu Z., Li D., Song L., Liu Y., Yu M., Zhang M., Shahidi F. (2020). Effects of proteolysis and oxidation on mechanical properties of sea cucumber (Stichopus japonicus) during thermal processing and storage and their control. Food Chemistry.

[bb0070] Nie S., Li L., Wang Y., Wu Y., Li C., Chen S., Wei Y. (2022). Discrimination and characterization of volatile organic compound fingerprints during sea bass (Lateolabrax japonicas) fermentation by combining GC-IMS and GC-MS. Food Bioscience.

[bb0075] Pangestuti R., Arifin Z. (2018). Medicinal and health benefit effects of functional sea cucumbers. Journal of Traditional and Complementary Medicine.

[bb0085] Sun Y., Fu J., Zhang E., Dong L., Cui X., Sun Y., Xu X. (2023). Fingerprint analysis of volatile flavor compounds in Crassostrea gigas of different ploidy and gender under high-temperature incubation. Molecules.

[bb0090] Wang M., Yang J., Zhao Q., Zhang K., Su C. (2019). Research Progress on flavor compounds and microorganisms of Maotai flavor baijiu. Journal of Food Science.

[bb0095] Wang Y., Tang X., Luan J., Zhu W., Xu Y., Yi S., Li X. (2022). Effects of ultrasound pretreatment at different powers on flavor characteristics of enzymatic hydrolysates of cod (Gadus macrocephalus) head. Food Research International.

[bb0100] Wei H., Wei Y., Qiu X., Yang S., Chen F., Ni H., Li Q. (2023). Comparison of potent odorants in raw and cooked mildly salted large yellow croaker using odor-active value calculation and omission test: Understanding the role of cooking method. Food Chemistry.

[bb0105] Xie J., Wang L., Deng Y., Yuan H., Zhu J., Jiang Y., Yang Y. (2023). Characterization of the key odorants in floral aroma green tea based on GC-E-nose, GC-IMS, GC-MS and aroma recombination and investigation of the dynamic changes and aroma formation during processing. Food Chemistry.

[bb0110] Xing L., Sun L., Liu S., Zhang L., Yang H. (2021). Comparative metabolomic analysis of the body wall from four varieties of the sea cucumber Apostichopus japonicus. Food Chemistry.

[bb0115] Xu C., Zhang R., Wen Z. (2018). Bioactive compounds and biological functions of sea cucumbers as potential functional foods. Journal of Functional Foods.

[bb0120] Xu X., Wu B., Zhao W., Pang X., Lao F., Liao X., Wu J. (2020). Correlation between autochthonous microbial communities and key odorants during the fermentation of red pepper (Capsicum annuum L.). Food Microbiology.

[bb0125] Xu Y., Bi S., Niu X., Chen Y., Liu Y., Zhou Q. (2023). Comparison of aroma active compounds in cold-and hot-pressed walnut oil by comprehensive two-dimensional gas chromatography-olfactory-mass spectrometry and headspace-gas chromatography-ion mobility spectrometry. Food Research International.

[bb0130] Yang D., Wu G., Li P., Qi X., Zhang H., Wang X., Jin Q. (2020). The effect of fatty acid composition on the oil absorption behavior and surface morphology of fried potato sticks via LF-NMR, MRI, and SEM. Food Chemistry: X.

[bb0135] Yang P., Wang H., Cao Q., Song H., Xu Y., Lin Y. (2023). Aroma-active compounds related to Maillard reaction during roasting in Wuyi rock tea. Journal of Food Composition and Analysis.

[bb0140] Yin P., Jia A., Heimann K., Zhang M., Liu X., Zhang W., Liu C. (2020). Hot water pretreatment-induced significant metabolite changes in the sea cucumber Apostichopus japonicus. Food Chemistry.

[bb0145] Zapana F., de Bruijn J., Vidal L., Melín P., González M.E., Cabrera G., Bórquez R. (2020). Physical, chemical and nutritional characteristics of puffed quinoa. International Journal of Food Science & Technology.

[bb0150] Zeng X., Liu J., Dong H., Bai W., Yu L., Li X. (2020). Variations of volatile flavour compounds in Cordyceps militaris chicken soup after enzymolysis pretreatment by SPME combined with GC-MS, GC× GC-TOF MS and GC-IMS. International Journal of Food Science & Technology.

[bb0155] Zhang H., Geng Y., Qin L., Dong X., Xu X., Du M., Dong L. (2018). Characterization of volatile compounds in different dried sea cucumber cultivars. Journal of Food Measurement and Characterization.

[bb0160] Zhang L., Peterson D.G. (2018). Identification of bitter compounds in extruded corn puffed products. Food Chemistry.

[bb0165] Zhao Y., Xue C., Zhang T., Wang Y. (2018). Saponins from sea cucumber and their biological activities. Journal of Agricultural and Food Chemistry.

[bb0170] Zheng X., Ji H., Zhang D., Zhang Z., Liu S., Song W. (2022). The identification of three phospholipid species roles on the aroma formation of hot-air-dried shrimp (Litopenaeus vannamei) by gas chromatography-ion mobility spectrometry and gas chromatography- mass spectrometry. Food Research International.

[bb0175] Zhou X., Chong Y., Ding Y., Gu S., Liu L. (2016). Determination of the effects of different washing processes on aroma characteristics in silver carp mince by MMSE–GC–MS, e-nose and sensory evaluation. Food Chemistry.

