# Supplementary material for: The dynamic changes of flavor characteristics of sea cucumber (Apostichopus japonicus) during puffing revealed by GC–MS combined with HS-GC-IMS
Source: Food Chem X. 2024 Aug 2;23:101709. doi: 10.1016/j.fochx.2024.101709 (PMC11357860; doi:10.1016/j.fochx.2024.101709)
Supplement: Supplementary file 1 — Supplementary material [file mmc1.docx]

# Submit online to Food Chemistry:X

The ​Dynamic Changes of Flavor Characteristics of Sea Cucumber (*Apostichopus japonicus*) during Puffing Revealed by GC-MS Combined with HS-GC-IMS

Xiaoqing Miao ^a^, Shuang Li ^a^, Yang Liu ^a^, Jing Li ^a^, Xiuping Dong ^a^，Ming Du^a^, Pengfei Jiang ^a,*^

^a^ SKL of Marine Food Processing & Safety Control, National Engineering Research Center of Seafood, School of Food Science and Technology, Dalian Polytechnic University, Dalian 116034, China

* Corresponding author:

Professor Pengfei Jiang

No. 1 Qinggongyuan, Ganjingzi District,

National Engineering Research Center of Seafood, School of Food Science and Technology, Dalian Polytechnic University, Dalian, P.R. China, 116034

Tel.: +86 411 86323262

Fax: +86 411 86323262

E-mail address: [jiangpf@dlpu.edu.cn](mailto:jiangpf@dlpu.edu.cn)

**Supporting Information**

**Table S1**

Sensory scoring rules for sea cucumbers under different puffing conditions

| Score | 0-3 | 4-6 | 7-9 |
| --- | --- | --- | --- |
| Color | Uneven color | Uniform color | Uniform color |
| Taste | Hard to chew | Harder and barely chewable | Crisp and chewable |
| Morphology | Appearance cracks are obvious | Appearance without obvious cracks | Appearance of flat and no cracks |
| Umami | No umami flavor | Mild umami flavor | Obvious umami flavor |
| Fishy | Strong and irritating fishy flavor | Mild fishy odor | No obvious fishy odor |

**Table S2**

Relative contents of VOCs in sea cucumbers under different puffing conditions

| Compound | Relative content / % | | | | | |
| --- | --- | --- | --- | --- | --- | --- |
|  | 210A | 210B | 230A | 230B | 250A | 250B |
| 1-Nonanol | 0.43±0.12b | 0.65±0.04a | 0.61±0.06a | 0.62±0.05a | 0.58±0.01a | 0.57±0.05a |
| 1-Octanol | 1.07±0.40b | 1.57±0.18a | 1.5±0.15ab | 1.65±0.06a | 1.35±0.06ab | 1.43±0.07ab |
| Propanoic acid | 0.21±0.03b | 0.22±0.01b | 0.22±0.00b | 0.24±0.01ab | 0.24±0.01ab | 0.28±0.04a |
| 1H-Pyrrole | 0.26±0.02c | 0.38±0.02ab | 0.34±0.02bc | 0.37±0.03ab | 0.41±0.03ab | 0.47±0.09a |
| Benzaldehyde(M) | 0.90±0.02f | 1.25±0.04e | 1.32±0.07d | 1.39±0.02c | 1.71±0.04b | 1.98±0.03a |
| Benzaldehyde(D) | 0.24±0.05d | 0.34±0.03c | 0.34±0.01c | 0.38±0.02c | 0.48±0.05b | 0.60±0.02a |
| Acetic acid(M) | 3.01±0.03a | 2.76±0.06b | 2.78±0.04b | 2.69±0.03b | 2.45±0.03c | 2.52±0.12c |
| Acetic acid(D) | 0.98±0.04a | 0.88±0.03b | 0.85±0.03bc | 0.84±0.02bc | 0.79±0.03c | 0.86±0.04bc |
| 1-Heptanol | 0.27±0.05a | 0.28±0.01a | 0.28±0.01a | 0.28±0.00a | 0.29±0.02a | 0.29±0.02a |
| 2-Nonanone(M) | 0.63±0.06b | 0.79±0.05c | 0.73±0.02c | 0.81±0.02a | 0.79±0.02c | 0.88±0.04a |
| 2-Nonanone(D) | 0.12±0.02d | 0.18±0.04bc | 0.16±0.01cd | 0.19±0.01bc | 0.23±0.03ab | 0.24±0.03a |
| 1-Hexanol | 0.20±0.01a | 0.20±0.01a | 0.20±0.01a | 0.22±0.01a | 0.22±0.01a | 0.21±0.00a |
| (Z)-2-Penten-1-ol | 0.12±0.01a | 0.12±0.00a | 0.13±0.01a | 0.13±0.00a | 0.12±0.01a | 0.12±0.02a |
| 2-Methyl-2-hepten-6-one | 0.36±0.01c | 0.4±0.02bc | 0.43±0.01bc | 0.46±0.03b | 0.46±0.02b | 0.59±0.07a |
| (E)-2-Heptenal | 0.24±0.03ab | 0.21±0.00b | 0.21±0.01b | 0.23±0.01ab | 0.26±0.01a | 0.21±0.00b |
| 1-Octanal(M) | 1.68±0.02a | 1.45±0.03c | 1.58±0.06b | 1.46±0.03c | 1.24±0.03d | 1.19±0.05d |
| 1-Octanal(D) | 3.54±0.04a | 2.54±0.09c | 2.92±0.16b | 2.60±0.20c | 2.14±0.04d | 1.70±0.06e |
| 2-Octanone(M) | 0.56±0.01d | 0.74±0.00b | 0.69±0.01c | 0.77±0.01b | 0.75±0.01b | 0.82±0.02a |
| 2-Octanone(D) | 0.75±0.08d | 1.28±0.15bc | 1.09±0.06c | 1.39±0.03ab | 1.54±0.09a | 1.59±0.17a |
| 3-Hydroxy-2-butanone | 0.10±0.02e | 0.16±0.01c | 0.14±0.01d | 0.17±0.00c | 0.27±0.01b | 0.36±0.00a |
| 1-Penten-3-ol | 1.69±0.02a | 1.63±0.04a | 1.64±0.04a | 1.57±0.02b | 1.42±0.02c | 1.38±0.03c |
| 4-Hexen-3-one(M) | 0.07±0.02a | 0.11±0.02c | 0.09±0.01cd | 0.12±0.01c | 0.19±0.02b | 0.35±0.01a |
| 4-Hexen-3-one(D) | 0.07±0.01b | 0.08±0.00b | 0.07±0.00b | 0.07±0.00b | 0.09±0.00b | 0.18±0.01a |
| (Z)-2-Methylpent-2-enal(M) | 0.85±0.05b | 0.86±0.02ab | 0.91±0.03a | 0.87±0.01ab | 0.81±0.01b | 0.83±0.02b |
| (Z)-2-Methylpent-2-enal(D) | 0.52±0.06c | 0.67±0.04b | 0.7±0.04b | 0.7±0.03b | 0.81±0.04a | 0.83±0.05a |
| 1-Butanol | 0.27±0.03b | 0.28±0.01b | 0.30±0.01b | 0.26±0.00b | 0.27±0.01b | 0.33±0.01a |
| (E)-2-Pentenal(M) | 0.94±0.01c | 1.00±0.05bc | 0.92±0.01c | 0.95±0.02c | 1.07±0.03ab | 1.12±0.07a |
| (E)-2-Pentenal(D) | 0.60±0.02b | 0.62±0.04b | 0.58±0.05b | 0.59±0.02b | 0.72±0.03a | 0.62±0.04b |
| 1-Hexanal(M) | 2.51±0.04a | 2.09±0.08c | 2.23±0.03b | 2.03±0.06c | 1.76±0.01d | 1.61±0.10e |
| 1-Hexanal(D) | 2.16±0.45a | 1.67±0.21a | 2.09±0.21a | 1.89±0.17a | 2.04±0.20a | 1.12±0.07b |
| 2-Hexanone(M) | 0.38±0.02c | 0.46±0.03b | 0.41±0.01c | 0.43±0.01bc | 0.38±0.02c | 0.51±0.03a |
| 2-Hexanone(D) | 0.44±0.04d | 0.79±0.09b | 0.68±0.03c | 0.85±0.06b | 1.06±0.03a | 0.97±0.05a |
| 3-Hexanone(M) | 0.44±0.00b | 0.53±0.01a | 0.46±0.03b | 0.52±0.01a | 0.46±0.01a | 0.51±0.03b |
| 3-Hexanone(D) | 0.18±0.01c | 0.32±0.01b | 0.23±0.02c | 0.32±0.02b | 0.31±0.03b | 0.42±0.06a |
| 2,3-Pentadione | 1.22±0.22c | 1.35±0.06c | 1.52±0.12bc | 1.36±0.04c | 1.85±0.12a | 1.72±0.17ab |
| Butanoic acid ethyl ester(M) | 0.55±0.04ab | 0.55±0.05ab | 0.63±0.03a | 0.51±0.04b | 0.55±0.03ab | 0.55±0.02ab |
| Butanoic acid ethyl ester(D) | 0.65±0.12c | 0.84±0.10bc | 0.99±0.15b | 0.79±0.11bc | 1.31±0.11a | 1.48±0.09a |
| 1-Pentanal(M) | 1.29±0.02a | 0.98±0.03c | 1.05±0.04b | 0.92±0.01d | 0.73±0.02e | 0.70±0.03e |
| 1-Pentanal(D) | 3.96±0.12a | 2.49±0.17c | 2.98±0.04b | 2.48±0.10c | 1.85±0.06d | 1.51±0.03e |
| 2-Pentanone | 1.76±0.01e | 2.70±0.09c | 2.22±0.04d | 2.63±0.12c | 3.10±0.08b | 3.47±0.08a |
| Ethanol | 0.87±0.02a | 0.62±0.03c | 0.70±0.01b | 0.56±0.01d | 0.40±0.02e | 0.42±0.05e |
| 2-Butanone | 4.06±0.19e | 5.47±0.24c | 5.04±0.08d | 5.51±0.03c | 5.91±0.09b | 6.44±0.08a |
| 3-Methyl butanal | 1.67±0.02a | 1.24±0.07c | 1.38±0.02b | 1.22±0.07c | 0.95±0.02d | 0.83±0.06e |
| Acetic acid ethyl ester | 0.63±0.18c | 0.91±0.12ab | 1.08±0.15a | 0.71±0.14c | 1.20±0.10a | 1.17±0.03a |
| Butanal | 1.9±0.04a | 1.41±0.09c | 1.57±0.04b | 1.42±0.05c | 1.03±0.01d | 0.85±0.02e |
| 2-Methylfuran | 0.95±0.01b | 0.92±0.04b | 0.86±0.04b | 0.90±0.01b | 0.94±0.02b | 1.24±0.07a |
| 2-Propanone | 8.80±0.18c | 9.63±0.11a | 9.29±0.12b | 9.37±0.02b | 9.57±0.01a | 9.75±0.10a |
| Propanal | 2.02±0.15a | 1.78±0.12bc | 1.85±0.04ab | 1.66±0.10bc | 1.59±0.04c | 1.56±0.08c |
| Pyrazine | 0.07±0.00b | 0.08±0.00b | 0.09±0.01a | 0.09±0.00a | 0.09±0.01a | 0.09±0.01a |
| Butyl propanoate | 0.10±0.00c | 0.12±0.01b | 0.13±0.01ab | 0.11±0.00b | 0.13±0.00ab | 0.14±0.01a |
| Butanoic acid butyl ester | 0.26±0.03a | 0.23±0.01a | 0.28±0.02a | 0.25±0.02a | 0.27±0.02a | 0.26±0.02a |
| 1-Nonanal(M) | 1.82±0.15a | 1.57±0.03c | 1.67±0.08ab | 1.57±0.02c | 1.69±0.03ab | 1.54±0.07c |
| 1-Nonanal(D) | 0.31±0.08ab | 0.22±0.01c | 0.27±0.04ab | 0.24±0.03ab | 0.35±0.04a | 0.28±0.04ab |
| 1-Pentanol(M) | 0.61±0.02abc | 0.6±0.02bc | 0.66±0.02ab | 0.62±0.01abc | 0.64±0.03ab | 0.58±0.02d |
| 1-Pentanol(D) | 0.27±0.01a | 0.26±0.02ab | 0.24±0.01c | 0.24±0.01c | 0.26±0.00ab | 0.23±0.01c |
| p-Cymene(M) | 0.66±0.01b | 0.73±0.02a | 0.71±0.04a | 0.73±0.01a | 0.65±0.03b | 0.73±0.01a |
| p-Cymene(D) | 0.24±0.01d | 0.39±0.04c | 0.36±0.02c | 0.43±0.01bc | 0.49±0.03b | 0.65±0.07a |
| (E)-2-Hexenal(M) | 0.70±0.00a | 0.67±0.02b | 0.71±0.01a | 0.70±0.00a | 0.66±0.01b | 0.6±0.00c |
| (E)-2-Hexenal(D) | 0.25±0.01bc | 0.25±0.03bc | 0.26±0.02abc | 0.28±0.01a | 0.3±0.01a | 0.22±0.02c |
| Heptaldehyde(M) | 0.98±0.03a | 0.75±0.08b | 0.8±0.04b | 0.69±0.05b | 0.56±0.04c | 0.56±0.05c |
| Heptaldehyde(D) | 1.38±0.03a | 0.92±0.06c | 1.02±0.02b | 0.92±0.05c | 0.83±0.03d | 0.76±0.01e |
| 2-Heptanone(M) | 0.99±0.04ab | 0.97±0.03ab | 1.01±0.02a | 0.95±0.01c | 0.87±0.02d | 0.88±0.03d |
| 2-Heptanone(D) | 4.72±0.07b | 5.56±0.23a | 5.36±0.08a | 5.72±0.12a | 5.73±0.17a | 5.45±0.27a |
| 1-Penten-3-one(M) | 0.18±0.03a | 0.12±0.01b | 0.11±0.01b | 0.12±0.01b | 0.10±0.01b | 0.10±0.02b |
| 1-Penten-3-one(D) | 0.09±0.03a | 0.04±0.00bc | 0.03±0.01d | 0.05±0.01bc | 0.06±0.01abc | 0.07±0.01ab |
| Dimethyl sulfide(M) | 1.05±0.05a | 0.78±0.05bc | 0.84±0.02b | 0.71±0.03c | 0.52±0.01d | 0.54±0.05d |
| Dimethyl sulfide(D) | 3.86±0.18a | 3.43±0.26ab | 3.00±0.35bc | 3.28±0.21b | 2.66±0.25cd | 2.37±0.10d |
| Acetaldehyde(M) | 0.35±0.02a | 0.32±0.04ab | 0.33±0.01ab | 0.32±0.01ab | 0.29±0.02b | 0.29±0.00b |
| Acetaldehyde(D) | 0.62±0.05d | 0.75±0.03c | 0.79±0.05c | 0.77±0.03c | 1.02±0.03b | 1.09±0.02a |
| Methyl 2-furoate | 0.08±0.02d | 0.13±0.02c | 0.13±0.01c | 0.13±0.01c | 0.24±0.02b | 0.30±0.02a |
| 1-Octen-3-ol | 0.59±0.07b | 0.58±0.01b | 0.62±0.03ab | 0.65±0.02ab | 0.70±0.04a | 0.66±0.03ab |
| 2-Decanone | 0.44±0.06a | 0.54±0.10a | 0.42±0.04a | 0.49±0.03a | 0.47±0.15a | 0.40±0.04a |
| 2,3,5-Trimethylpyrazine | 0.07±0.01b | 0.1±0.02a | 0.09±0.00a | 0.11±0.00a | 0.10±0.01a | 0.09±0.01a |
| (E)-2-Octenal | 0.33±0.05b | 0.26±0.01b | 0.27±0.00ab | 0.27±0.01ab | 0.33±0.02a | 0.31±0.01ab |
| 2,3-Dimethylpyrazine | 0.10±0.01d | 0.13±0.01c | 0.13±0.00c | 0.13±0.01c | 0.16±0.01b | 0.19±0.01a |
| 4-Methyl pentanol | 0.10±0.01a | 0.12±0.03a | 0.10±0.01a | 0.13±0.03a | 0.13±0.04a | 0.10±0.01a |
| 2,6-Dimethylpyridine | 0.13±0.01d | 0.23±0.03c | 0.23±0.03c | 0.25±0.04c | 0.43±0.09b | 0.53±0.03a |
| Ethyl 2-oxopropanoate | 3.41±0.07a | 2.79±0.04d | 3.21±0.01b | 2.97±0.05c | 2.72±0.03d | 2.42±0.05e |
| 2-Pentyl furan | 1.06±0.05a | 1.15±0.25a | 0.91±0.09a | 1.31±0.15a | 1.23±0.24a | 0.97±0.02a |
| 2-Ethyl furan | 3.94±0.02a | 2.97±0.42b | 2.88±0.12b | 2.80±0.15b | 1.81±0.15c | 1.52±0.04c |
| Acetic acid butyl ester | 0.14±0.00c | 0.16±0.01bc | 0.16±0.01b | 0.15±0.00b | 0.20±0.01a | 0.21±0.01a |

**Table S3**

ROAV values and flavor description of VOCs in sea cucumbers under different puffing conditions

| Compound | Odour threshold  (μg/kg) | Flavor description | ROAV | | | | | |
| --- | --- | --- | --- | --- | --- | --- | --- | --- |
|  |  |  | 210A | 210B | 230A | 230B | 250A | 250B |
| 1-Nonanol | 18 | - | 0.146 | 0.256 | 0.266 | 0.260 | 0.305 | 0.329 |
| 1-Octanol | 110 | - | 0.059 | 0.102 | 0.107 | 0.113 | 0.116 | 0.135 |
| 1-Heptanol | 3 | - | 0.547 | 0.673 | 0.739 | 0.704 | 0.915 | 1.010 |
| 1-Hexanol | 5.6 | Oily, fruity | 0.217 | 0.258 | 0.280 | 0.294 | 0.375 | 0.381 |
| 1-Octen-3-ol | 1 | Mushroom, earthy flavor | 3.583 | 4.161 | 4.862 | 4.858 | 6.566 | 6.793 |
| 2-Nonanone | 5 | Grassy | 0.920 | 1.381 | 1.404 | 1.504 | 1.919 | 2.311 |
| 2-Octanone | 50 | Creamy, soapy | 0.161 | 0.288 | 0.279 | 0.324 | 0.432 | 0.498 |
| 3-Hydroxy-2-butanone | 14 | - | 0.045 | 0.084 | 0.079 | 0.091 | 0.181 | 0.265 |
| 2-Hexanone | 40 | Creamy, fruity | 0.126 | 0.223 | 0.213 | 0.240 | 0.340 | 0.382 |
| 3-Hexanone | 35 | Sweet, fruity, waxy | 0.108 | 0.173 | 0.154 | 0.182 | 0.208 | 0.272 |
| 2-Propanone | 832 | - | 0.065 | 0.082 | 0.087 | 0.085 | 0.109 | 0.121 |
| 1-Penten-3-one | 1.2 | Grassy | 1.359 | 0.928 | 0.954 | 1.057 | 1.293 | 1.480 |
| 2-Heptanone | 140 | - | 0.249 | 0.333 | 0.356 | 0.358 | 0.445 | 0.467 |
| 2-Decanone | 9 | Fruity, earthy and musty | 0.299 | 0.424 | 0.367 | 0.407 | 0.490 | 0.459 |
| 1-Octanal | 0.7 | Fatty, grassy, lemony | 45.605 | 40.686 | 50.270 | 43.524 | 45.591 | 42.502 |
| (E)-2-Octenal | 3 | Grassy, sweet, citrus | 0.666 | 0.627 | 0.706 | 0.684 | 1.031 | 1.056 |
| 1-Hexanal | 5 | Greasy flavor | 5.715 | 5.358 | 6.752 | 5.901 | 7.164 | 5.639 |
| 1-Pentanal | 12 | Almonds, malt | 2.673 | 2.056 | 2.626 | 2.129 | 2.029 | 1.908 |
| 3-Methyl butanal | 0.35 | Almonds, cocoa | 29.170 | 25.271 | 30.900 | 26.261 | 25.547 | 24.352 |
| Butanal | 7 | Banana, stimulating | 1.656 | 1.438 | 1.749 | 1.523 | 1.386 | 1.254 |
| Propanal | 15 | Cocoa, floral, nutty | 0.825 | 0.847 | 0.965 | 0.834 | 0.998 | 1.071 |
| 1-Nonanal(M) | 1.1 | Fishy, harry odor | 11.846 | 11.605 | 13.793 | 12.383 | 17.522 | 17.070 |
| (E)-2-Hexenal(M) | 17 | Lipo, grassy, fruity | 0.341 | 0.388 | 0.447 | 0.435 | 0.533 | 0.496 |
| Heptaldehyde(M) | 3 | Fishy, harry odor | 4.796 | 3.972 | 4.732 | 4.041 | 4.371 | 4.535 |
| Acetaldehyde(M) | 15 | - | 0.397 | 0.505 | 0.584 | 0.548 | 0.821 | 0.951 |
| Butanoic acid ethyl ester(M) | 20 | Sweet, fruity, floral | 0.366 | 0.496 | 0.633 | 0.492 | 0.880 | 1.048 |
| Acetic acid ethyl ester | 5 | Balsamic vinegar, fruit | 0.771 | 1.290 | 1.686 | 1.066 | 2.265 | 2.407 |
| Acetic acid butyl ester | 12 | Fruity, Sweet | 0.072 | 0.095 | 0.106 | 0.094 | 0.158 | 0.182 |
| p-Cymene(M) | 57 | - | 0.097 | 0.139 | 0.147 | 0.152 | 0.189 | 0.250 |
| Dimethyl sulfide(M) | 0.3 | Sulfur, onions, sweet corn | 100.000 | 100.000 | 100.000 | 100.000 | 100.000 | 100.000 |
| 2-Pentyl furan | 6 | Fruity, grassy, hammy | 0.981 | 1.364 | 1.189 | 1.643 | 1.942 | 1.669 |
| 2-Ethyl furan | 2.3 | caramelized aroma | 10.474 | 9.205 | 9.793 | 9.141 | 7.431 | 6.818 |
